# Environmental and anthropogenic drivers of watercress (*Nasturtium officinale*) communities in char-lands and water channels across the Swat River Basin: implication for conservation planning

**DOI:** 10.3389/fpls.2023.1225030

**Published:** 2023-09-27

**Authors:** Nasrullah Khan, Rafi Ullah, Mohammad K. Okla, Mostafa A. Abdel-Maksoud, Ibrahim A. Saleh, Hashem A. Abu-Harirah, Tareq Nayef AlRamadneh, Hamada AbdElgawad

**Affiliations:** ^1^ Department of Botany, University of Malakand, Khyber Pakhtunkhwa, Pakistan; ^2^ Department of Botany and Microbiology, College of Science, King Saud University, Riyadh, Saudi Arabia; ^3^ Faculty of Science, Zarqa University, Zarqa, Jordan; ^4^ Department of Medical Labortory Sciences, Faculty of Allied Medical Sciences, Zarqa University, Zarqa, Jordan; ^5^ Integrated Molecular Plant Physiology Research, Department of Biology, Univeristy of Antwerp, Antwerp, Belgium

**Keywords:** physical environment, vegetation structure, anthropogenic factors, charland, *Nasturtium officinale*

## Abstract

Recent anthropogenic sources and excess usage have immensely threatened the communities and habitat ecology of this region’s medicinally and economically significant crops. Therefore, our study aims to evaluate the community structure and related environmental characteristics sustaining *Nasturtium officinale* communities along the river basin (RB) in Northwest Pakistan, using the clustering procedure (Ward’s method) and Redundancy analysis (RDA). From 340 phytosociological plots (34 × 10 = 340), we identified four ecologically distinct assemblages of *N. officinale* governed by different environmental and anthropogenic factors for the first time. The floristic structure shows the dominance of herbaceous (100%), native (77%), and annual (58.09%) species indicating relatively stable communities; however, the existence of the invasive plants (14%) is perturbing and may cause instability in the future, resulting in the replacement of herbaceous plant species. Likewise, we noticed apparent variations in the environmental factors, i.e., clay percentage (*p* = 3.1 × 10**^−5^**), silt and sand percentage (*p*< 0.05), organic matter (*p*< 0.001), phosphorus and potassium (*p*< 0.05), and heavy metals, i.e., Pb, Zn, and Cd (*p*< 0.05), indicating their dynamic role in maintaining the structure and composition of these ecologically distinct communities. RDA has also demonstrated the fundamental role of these factors in species–environment correlations and explained the geospatial variability and plants’ ecological amplitudes in the Swat River wetland ecosystem. We concluded from this study that *N. officinale* communities are relatively stable due to their rapid colonization; however, most recent high anthropogenic interventions especially overharvesting and sand mining activities, apart from natural enemies, water deficit, mega-droughts, and recent flood intensification due to climate change scenario, are robust future threats to these communities. Our research highlights the dire need for the sustainable uses and conservation of these critical communities for aesthetics, as food for aquatic macrobiota and humans, enhancing water quality, breeding habitat, fodder crop, and its most promising medicinal properties in the region.

## 1 Introduction

Biodiversity regulates the ecosystem functioning and stability and is essential for human survival and economic wellbeing (Singh, 2002; Htun et al., 2011; Biplab et al., 2017; Baboo et al., 2017). The loss of biodiversity is a major challenge faced by humans in maintaining the stability and functional sustainability of ecosystems (Hua et al., 2022; Bisht et al., 2023). Wetland ecosystems are threatened by anthropogenic activities (Bisht et al., 2022). Among these, poverty, human population, agricultural expansion and intensification, and infrastructure development have been suggested as major threats to biodiversity in the tropics (Davidar et al., 2010; Bargali et al., 2019; Bargali et al., 2022; Bisht et al., 2023). Wetland ecosystem and its vegetation provide a wide range of ecosystem goods and services to the inhabitants (Gosain et al., 2015; Fartyal et al., 2022). Overexploitation of natural resources has created a big gap between the demand and supply of the natural goods (Manral et al., 2020; Pandey et al., 2023).

The biodiversity patterns along environmental gradients are one of the fundamental questions and are the synthetic reflection of all different types of ecological research (Khan S. et al., 2011; Raduła et al., 2020; Yu et al., 2021). The foundation of biodiversity research is based on the patterns of plant species diversity and the ecological variables affecting these patterns (Nogués-Bravo et al., 2008; Johnson et al., 2015; Li et al., 2018). Because of its importance in predicting future community composition and species performance, plant species diversity has received significant attention (Ullah et al., 2022; Khan et al., 2020; Wang et al., 2008; Subedi et al., 2020). The preservation of ecosystem biodiversity is significantly aided by habitat diversity, where topographical factors have multidimensional and multiscale effects on the pattern of species diversity (De Leo et al., 2014). These factors are interconnected and indicators of several abiotic parameters, including geographic, topographic and soil nutrient flow (Twilley and Rivera-Monroy, 2005).

The spatial scale will likely determine how an organism affects its surroundings, including ecosystem services (Ze-Hao and Xin-Shi, 2000; Kremen et al., 2007). In recent research using worldwide field survey data, organism–environment linkages may change with geography (Grace and Anderson, 2016). Abiotic factors at the regional scale determines the species distribution, as the effects of biotic factors (species types) on their environment are typically more localized (van de Koppel et al., 2012). However, direct and indirect interaction of these factors ultimately constitute organism–environment relationship (Grace and Anderson, 2016). The influence of biota may have been overstated in most studies due to the prevalence of statistical techniques (Alsterberg et al., 2013; Grace and Anderson, 2016).

The edaphic and geographical elements are also important for the variety of plant species and their distribution in aquatic emergent (char-land and water channels) plant communities (Schlaepfer et al., 2012). According to Chao et al. (2006), the soil’s physicochemical characteristics and parent materials may impact the plants growing there and the species diversity. Various direct ecological factors at large scales, including soil texture, nutrient level, and soil hydraulic and metal concentration, influence the distribution and structure of plant communities and species biodiversity in emergent plant communities (Stella et al., 2013). However, the altitudinal gradient is the primary factor forming different mountain habitats and is one of the key elements determining the spatial patterns of species diversity in many regions (Bhattarai and Vetaas, 2003; Wang et al., 2003; Zhao et al., 2005; Jiang et al., 2007; Bhattarai et al., 2014; Subedi et al., 2020).

According to Mitsch and Gosselink (1993), the main factor affecting the distribution of plant species at the land–water interface in wetlands is the water regime. In addition, wetland vegetation’s spatial heterogeneity is influenced by several variables, such as soil composition, microclimate, and topography since it controls the water regime (Naiman et al., 2005; Rybicki and Landwehr, 2007; Watt et al., 2007). The ranges of aquatic plants have been estimated using a variety of methods (Mcfarland and Shafer, 2011; Santos et al., 2011; Orellana et al., 2012; Singh et al., 2020). Field observations and large-scale analyses may now be combined with advances in spatial technology like aerial photography and other remote sensing techniques (Vis et al., 2003) and potent geo-statistical tools like plant cover/density estimators (Pepe et al., 2018). However, owing to the biophysical limitations in the environment, it is important to accurately assess aquatic plant species’ spatial distribution and plant community composition and comprehend their interactions.

The recent anthropogenic activities and rapid urbanization have deliberately changed the natural wetland communities in many areas of the world, particularly in developing countries like Pakistan. These activities threatened the communities’ lies at the junction of terrestrial and aquatic ecosystems or on wetlands. For example, recently, Ali et al. (2022) reported different mining operations along the riparian vegetation that adversely affect the community composition and structure. Therefore, this study evaluated *Nasturtium officinale* (R. Br. In Aiton) communities’ floristic composition and structure along the River Swat using quantitative ecological parameters. The study aims to assess plant communities dominated by *N. officinale* and associated species, and to provide scientific basis on the following questions: (i) How do these natural bog-plant communities and species diversity at char-land and channels along the Swat River Basin (SRB) respond to ecological factors? (ii) How do different degrees of anthropogenic factors act in the instability perspective of *N. officinale* at the community-level? By analyzing the ecological data of community composition and structure, we anticipated specifying a valid scientific basis for species assemblage’s distribution and characterization besides its protection and management in the region.

## 2 Materials and methods

### 2.1 Study area

**Figure 1:**
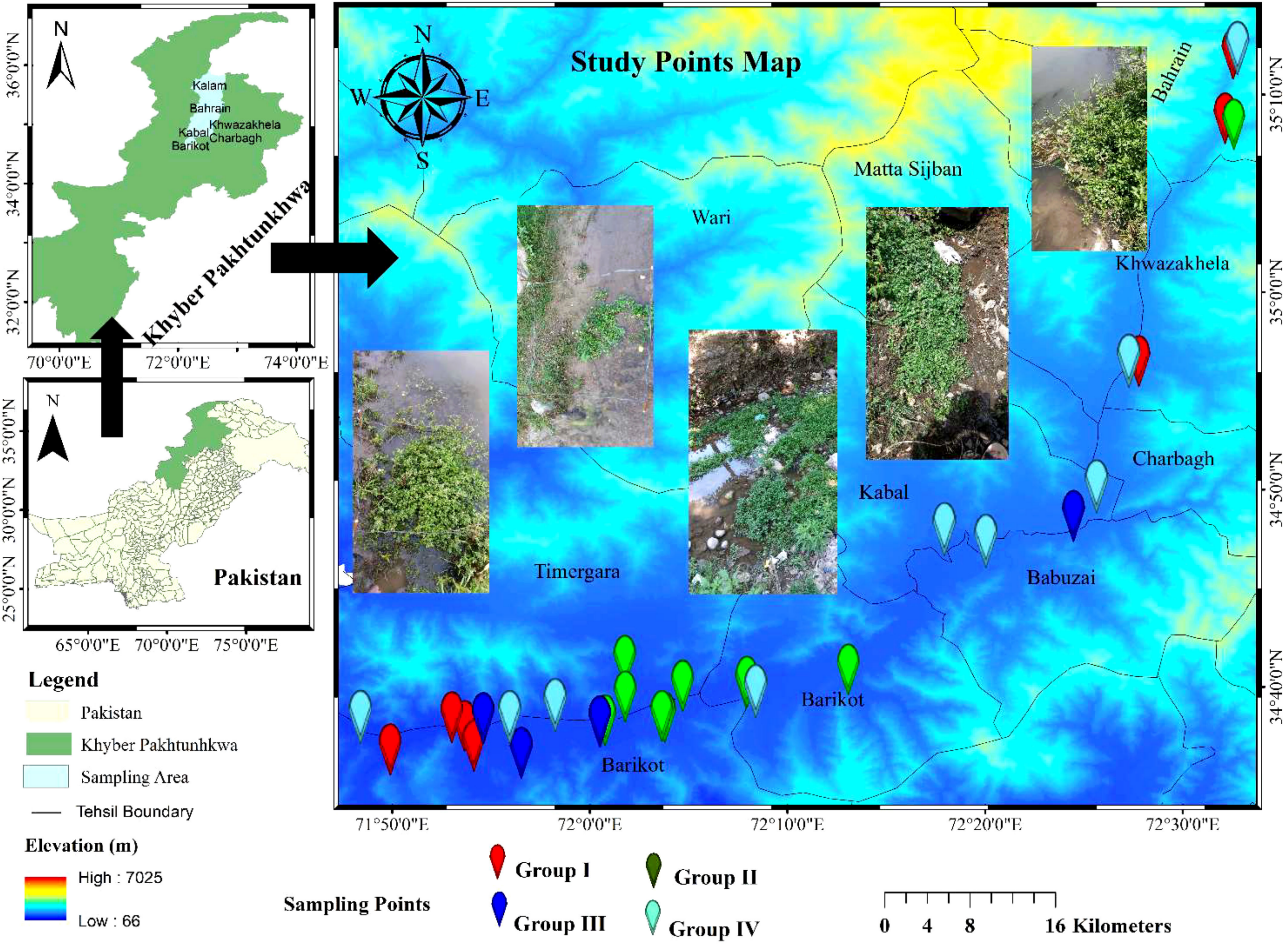
Map showing sampling points of the research area.

The bogs of River Swat were selected for sampling because of rich *Nasturtium officinale* communities, from where the residents and local sellers often collect vegetables for edible purposes. The study area is located at 35.22271134 North and 72.42581572 East, in the northern region of Khyber Pakhtunkhwa, Pakistan, spreading over 14,737 square kilometres area covered by hilly glaciers having snowfall and rain (**Figure 1**) (Farooq et al., 2018). River Swat flows through the districts of Swat, Malakand, and Dir Lower (Ahmad et al., 2015), having subtropical and temperate climates (Barinova et al., 2013). The average higher temperature in the summer may reach 41.9°C, while the average low in the winter can reach 0.8°C. The Pakistan Meteorological Department reported an annual average rainfall of 1,003 mm; the highest rainfall was recorded in February and March (162 mm). According to Nafees et al. (2008), relative humidity may drop as low as 40% in April and rise as high as 85% in July.

**Figure 2:**
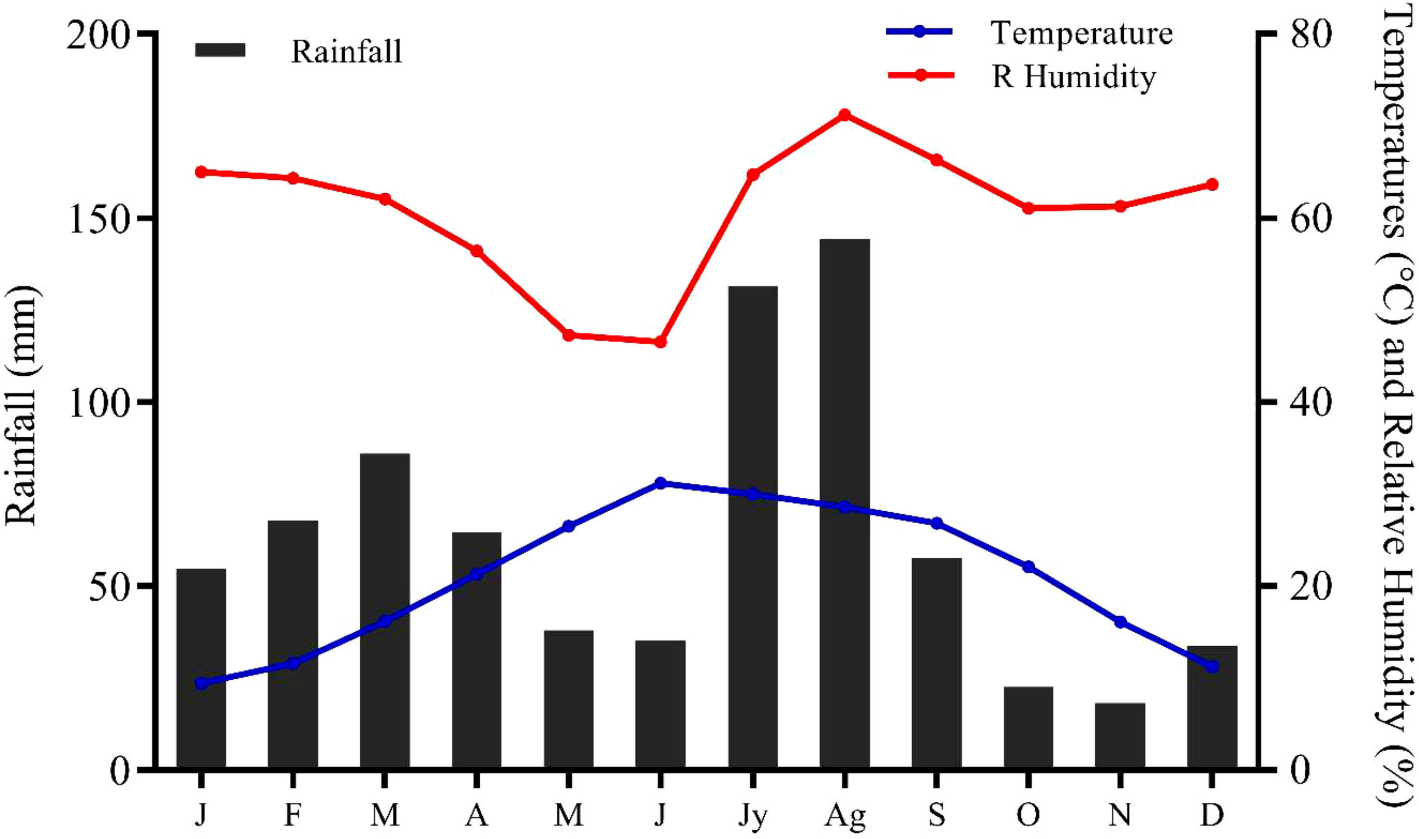
Important climate variables of the sampling sites. J, January; F, February; M, March; A, April; M, May; J, June; Jy, July; Ag, August; S, September; O, October; N, November; D, December.

Ali et al. (2018) report that the average monthly temperature in the study area is between 34.96°C and 1.36°C, and the average annual rainfall is between 384 to 639 mm (**Figure 2**). These factors maintain the local climate to comprehend the structure and composition of the vegetation that ultimately affects economic, social, and agricultural activities and hydrological characteristics (Deo and Şahin, 2015).

### 2.2 Field sampling protocols

In our field research, we randomly selected *N. officiale*-dominated stands at 34 distinct locations throughout five districts as part of routine trips to gather vegetation data (**Figure 1**). The entire area was represented by taking 340 phytosociological plots (34 × 10 = 340 plots) and carefully selected according to the structural and floristic homogeneity. The variations in plot size were considered following Morsdorf et al. (2010), where the plot sizes of 3–5 m^2^ were taken as standard for herb and shrub communities. The standard procedure for computing plant species densities, frequencies, covers, relative densities, relative frequencies, relative covers, and important values in each plot and sites was adopted following Curtis and Mcintosh (1950). To reduce edge effects, a 10-m buffer zone was removed from the stand’s boundaries following Martínez-Falcón et al. (2018). Nomenclature for plant taxa was taken from Flora of Pakistan (Ali, 2008), and binomials were given accordingly.

### 2.3 Soil data acquisition and analysis

In each stand, a soil sample of 3 kg was collected randomly from each stand’s middle and two opposing corners to characterize soil parameters. Since topsoil is often the most nutrient-rich part of the soil, soil samples were collected at a depth of 0–30 cm bulked and fully mixed (Bargali et al., 2018) to reduce heterogeneity (Morsdorf et al., 2010; Kamrani et al., 2011). The electrical conductivity and pH of a soil–water suspension (1:5) were measured in the field using a digital pH meter and an EC meter, respectively. The soil samples were allowed to air dry at room temperature and then passed through a sieve set at 2 mm. The samples were homogenized and packed in transparent polythene bags for subsequent processing (Chawla et al., 2008).

The soil’s physiochemical composition and textural parameters were determined by air-drying the samples and passing them through a 2-mm sieve per USDA recommendations (Taubner et al., 2009). Organic matter was assessed using the Walkley-Black method, while total and organic carbon was computed utilizing moist burning with chromic acid digestion after dry combustion (Nelson and Sommers, 1996). Total nitrogen was determined using the micro-Kjeldahl method, and soluble phosphorus (P^2+^) and exchangeable potassium (K^+^) were determined using the methods described by Yadav et al. (2002). Lime (calcium carbonate; %) was calculated geometrically, and CO_2_ evolution was tracked geometrically, following Gómez-Díaz et al. (2006). Following the methodology established by Saxton et al. (1986), we used an online calculator (https://www.nrcs.usda.gov) to determine the soil’s field capacity (FC), available water (AW), bulk density (BD), and wilting and saturation point (WSP). The distribution of soil textural characteristics, i.e., sand, silt, and clay percentage, was assessed using the hydrometer approach (Gangwar and Baskar, 2019).

### 2.4 Anthropogenic variables assessment and quantification of diversity indices

Semi-structured interviews were conducted with 200 respondents to identify the anthropogenic factors threatening the diversity of *N. officinale* communities. The respondents were selected based on their connection with the collection and sale of plant species. Following Kefalew et al. (2015), the respondents were categorized into two groups; i.e., category “A” includes 100 respondents selected randomly, and category B has 100 respondents that were chosen purposefully having past and present knowledge of the site where the plant species grows naturally. These respondents identified factors, i.e., mining factor, agricultural field disturbance (cultivation factors), grazing intensity, and over-harvesting that disturbed these communities severely. Of the respondents, 12 respondents, i.e., 5 from Category A and 7 from Category B, were excluded from the analysis as they do not respond to the questionnaire.

A six-point scale (0–5) was used to evaluate whether these anthropogenic factors are affected quantitatively. A plot was considered undisturbed if it obtained a score of 0, while a score of 5 was considered severely disturbed (Mligo, 2011). Accordingly, 0 denotes no disturbance, 1 represents a disruption of 0%–20% of the plot, 2 denotes a disruption of 21%–40% of the plot, 3 denotes a disruption of 41%–60% of the plot, 4 denotes a disruption of 61%–80% of the plot, and 5 denotes a disruption of 81–100% of the plot. Each type of disturbance was assessed separately in this semi-quantitative evaluation. The degrees of disturbance were evaluated based on the percentage of the given parameter persisting in a disturbing plot of 10 × 10 m. The point scale values were assessed according to Barry (2006) and Lewis et al. (2017) to describe various forms of anthropogenic disturbance.

In addition, the species richness and diversity indices, i.e., Shannon index *H*’ and Evenness index *E*, were used to describe group diversity (Maan et al., 2021).


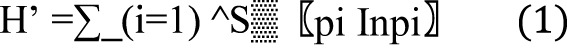



(2) E=H'/InS

where Σ = summation, S = species richness, pi = proportion of the species (i) to total number of species, In = natural logarithm.

### 2.5 Community data analysis and ordination

The species’ phytosociological data and relevant environmental parameters for the 34 stands were collected and compiled for statistical analysis. An important value index (IVI) based on comparative phytosociological features was calculated following Khan et al. (2013). Ward’s agglomerative technique (McCune, 1997) was used to evaluate the classification of vegetative communities by choosing Euclidean distance. Each species in the stand was then assigned a phytosociological group based on the numbers assigned to them. We examined recent studies to see whether a particular species might be taken as illustrative of a stand or group (Biondi et al., 2015; Rahman et al., 2022). The converted relative phytosociological values were used for numerical classification to determine the diagnostic species of a particular group. The Redundancy analysis (RDA) was used to determine the relationship between species IVI and environmental variables (20 soil and 4 topographic) of the *N. officinale*-dominated vegetation. The statistical *post-hoc* interpretation of the RDA ordination axes was analyzed using the Monte Carlo permutation test. The ordination bi-plot displayed the essential variables as vectors that determined the community structure and composition. Microsoft Excel 2010 and PC-ORD version 6.0 were used to conduct the statistical analysis of the quantitative vegetation and environmental data (Barua et al., 2020).

## 3 Results

### 3.1 Floristic characteristics

**Figure 3:**
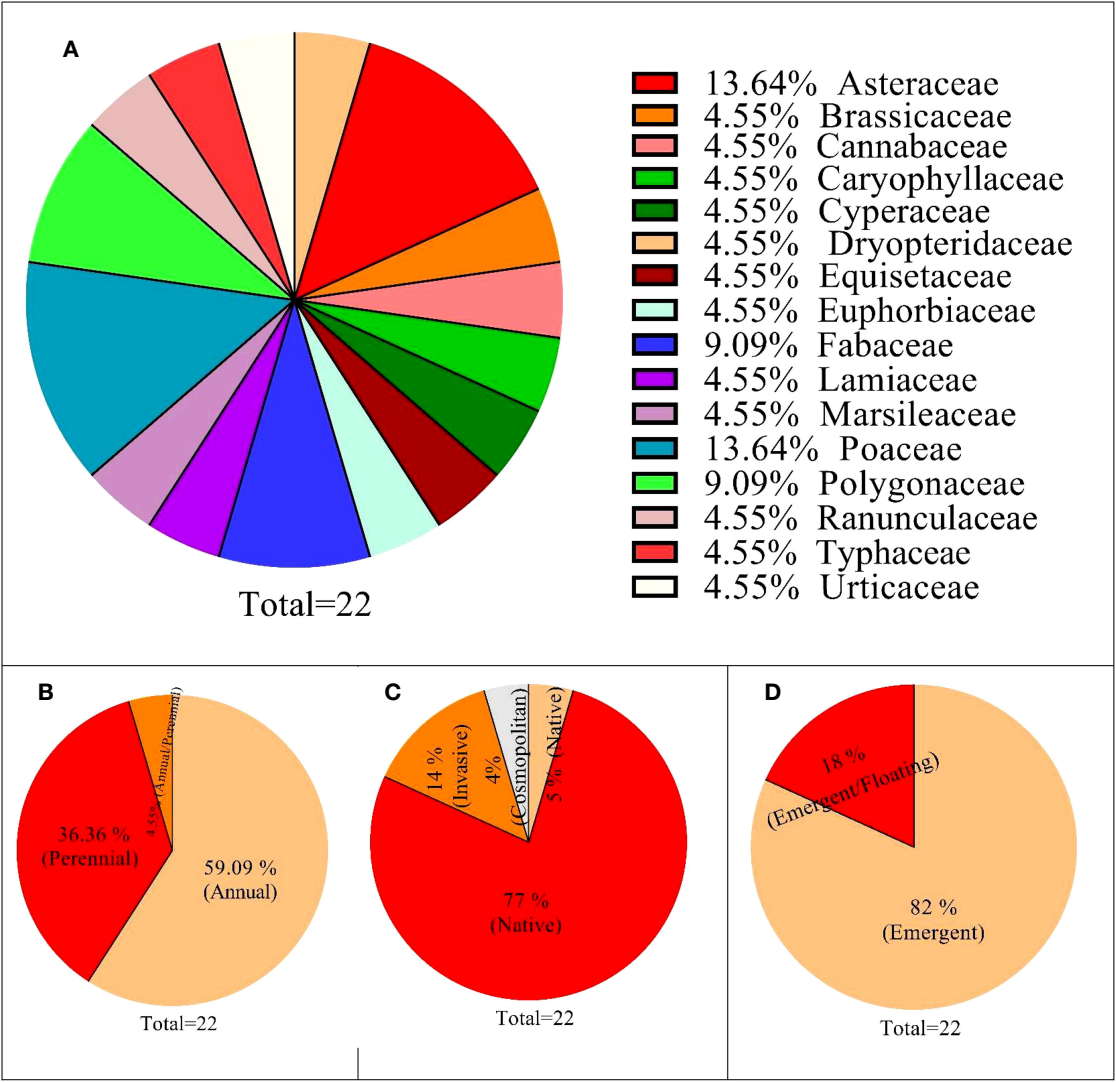
Floristic structure of *Nasturtium officinale* and associated species found in communities. **(A)** (family base distribution); **(B)** (life cycle), **(C)** (taxon), **(D)** (status in Pakistan), and **(E)** (aquatic habitat).

The floristics of vegetation consists of 22 species dominated by *N. officinale*, where all the species were either emergent or floating aquatic habitats indicating a wetland community. The species belongs to 16 families; Poaceae and Asteraceae were represented by three species each, followed by Polygonaceae and Fabaceae (two species each). The remaining 12 families were monospecific (**Figure 3**), where herbaceous plants were evident (100%) in the floristic composition, with 13 species having annual life cycles, 8 perennials, and 1 having both (**Table A1**). Similarly, based on status, 17 species were classified as native to Pakistan, 3 as invasive, and 1 each for cosmopolitan and naturalized. In addition, based on aquatic habitat, 18 species were emergent, and 4 were emergent/floating (**Figure 3**).

### 3.2 Vegetation classification and structural attributes

**Figure 4:**
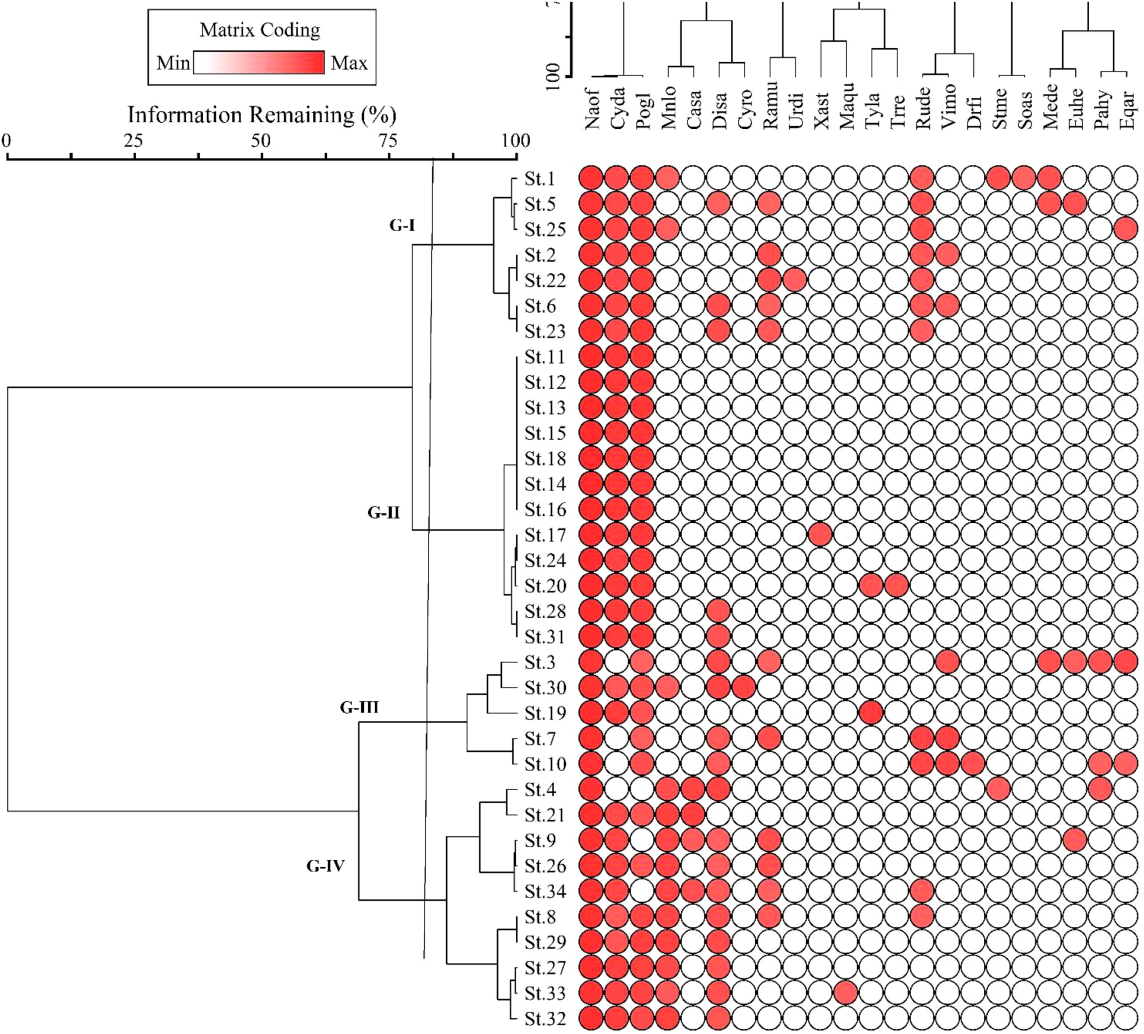
Two-way cluster dendrogram of *Nasturtium officinale* communities along the Swat River catchments. Plant species acronyms are the same as that in **Table 1**.

The two-way cluster dendrogram indicates four vegetation groups showing stands on one side and species distributed in these groups on the other (**Figure 4**). Similarly, the red dots indicated the intensity of the species’ IVI, i.e., its increase or decrease in the studied stands. Based on the number of stands, Group II was the largest, including 13 stands in the middle of the dendrogram. Group III was the smallest, having five stands of *N*. *officinale*-dominated vegetation. Group I has seven stands with 14 species and is considered the third largest community of vegetation dominated by *N. officinale* (42.9 ± 1.0), followed by *Polygonum glabrum* (19.4 ± 0.5) and *Cynodon dactylon* (13.4 ± 0.5). In comparison, the remaining 11 species of this group have a mean IVI of less than 10 as presented in **Table 1**. Group II has 13 stands with seven species and was considered the largest group with low species richness. The dominant species of the group was *N*. *officinale* (53.4 ± 0.4), having co-dominance of *Cynodon dactylon* (31.8 ± 2.5).

**Table 1:** Importance values index (IVI) of different plant species associated with *Nasturtium officinale* in different vegetation groups along the Swat River catchments.

Species Binomial	Acronyms	Group I	Group II	Group III	Group IV
		M ± SE	M ± SE	M ± SE	M ± SE
*Nasturtium officinale* W.T. Aiton	Naof	42.9 ± 1.0	53.4 ± 0.4	42.5 ± 1.4	45.0 ± 0.5
*Cannabis sativa* L.	Casa	*-	*-	3.4 ± 3.4	3.6 ± 2.1
*Cynodon dactylon* (L.) Pers	Cyda	13.4 ± 0.5	31.8 ± 2.5	1.1 ± 1.1	6.26 ± 1.1
*Cyperus rotundus* L.	Cyro	*-	*-	3.4 ± 3.4	*-
*Digitaria sanguinalis* (L.) scop.	Disa	3.4 ± 1.7	1.03 ± 0.7	11.8 ± 2.7	5.7 ± 0.9
*Dryopteris filix-mas* (L.) Schott	Drfi	*-	*-	1.5 ± 1.5	*-
*Equisetum arvense* L.	Eqar	0.80 ± 0.8	*-	3.6 ± 2.7	*-
*Euphorbia helioscopia* L.	Euhe	0.99 ± 0.9	*-	0.90 ± 0.9	0.5 ± 0.5
*Marsilea quadrifolia* L.	Maqu	*-	*-	*-	0.52 ± 0.5
*Medicago denticulata* Willd.	Mede	2.0 ± 1.3	*-	1.5 ± 1.5	*-
*Mentha longfolia* (L.) Huds.	Melo	1.2 ± 0.81	*-	2.9 ± 1.9	16.9 ± 0.9
*Parthenium hysterophorus* L.	Pahy	*-	*-	3.2 ± 1.4	*-
*Polygonum glabrum* Willd.	Poba	19.4 ± 0.5	14.6 ± 1.3	5.4 ± 1.7	3.2 ± 1.4
*Ranunculus muricatus* L.,	Ramu	4.8 ± 1.5	*-	2.7 ± 1.9	10.3 ± 2.5
*Rumex dentatus* L.	Rude	6.2 ± 0.7	*-	6.4 ± 3.9	1.03 ± 0.6
*Sonchus asper* (L.) Hill	Soas	0.62 ± 0.6	*-	*-	*-
*Stellaria media* (L.) Vill.	Stme	1.2 ± 1.2	*-	0.95 ± 0.9	*-
*Trifolium repens* L.	Trre	*-	0.51 ± 0.51	*-	*-
*Typha latifolia* L.,	Tyla	*-	2.13 ± 1.65	*-	*-
*Urtica dioica* L.	Utdi	0.67 ± 0.6	*-	*-	*-
*Vicia monantha* Retz.	Vimo	1.3 ± 0.8	*-	7.8 ± 3.6	*-
*Xanthium strumarium* L.	Xast	*-	0.51 ± 0.51	*-	*-
∑		14	7	16	10

∑, summation; M, mean; SE, standard error; *species absence in group.

Group III consists of five stands with 16 species dominated by *N. officinale* (42.5 ± 1.4) while the co-dominant species was *Digitaria sanguinalis* (11.8 ± 2.7). The important associated species of the group include *Vicia monantha* (7.8 ± 3.65), *Rumex dentatus* (6.4 ± 3.9), and *Polygonum glabrum* (5.4 ± 1.7). Similarly, Group IV consisted of 9 stands with 10 species and was considered the second largest community dominated by *N officinale* (45.0 ± 0.5), with *Mentha longfolia* (16.9 ± 0.9) being the co-dominant species. The other important species of the group are *Ranunculus muricatus* (10.3 ± 2.5) and *Cynodon dactylon* (6.26 ± 1.1).

Density/hectare and cover/hectare of *N. officinale*, and associated species (**Table A2**), revealed that, in Group I, the density of *N. officinale* is 46,243 ± 4,119 individuals/hectare. At the same time, the co-dominant species was *C*. *dactylon* (18,730 ± 2,316). In Group II, *N*. *officinale* has a density/ha of 57,094 ± 2,469 followed by *C*. *dactylon* (13,162 ± 1,939). Similarly, Group III is the largest community by the number of species, including *N*. *officinale*, with a density/hectare of 47,555 ± 7,702. However, the co-dominant species was *D*. *sanguinalis* (10,222 ± 3,723) while Group IV included 10 species having *C. dactylon* (11,851 ± 2,051) as the co-dominant species. Similarly, the same patterns have been followed by cover/hectare of *N. officinale* and the remaining co-dominant species.

### 3.3 Analysis of environmental and anthropogenic variables

The results of associated soil variables showed a sandy or silty loamy soil having significant variation in sand and clay particles (*p*< 0.05). In contrast, Groups II and IV have high contents of silt and sand (**Table 2**). The soil pH was basic, showing non-significant variation, while the electrical conductivity of Group III was higher (0.3 ± 0.05) than others. Similarly, total dissolved solids were high in Groups III and II, i.e., 0.09 ± 0.005 and 0.09 ± 0.01, respectively, complementing the electrical conductivity results. The soil nutrients, Group IV, had higher contents of organic matter (0.97 ± 0.02), and varied significantly (*p*< 0.05) within the vegetation groups, while the nitrogen content of all the community groups was the same, i.e., 0.04 ± 0.002. In addition, the essential nutrients, i.e., phosphorus and potassium contents (mg kg^−1^) of Group II (35.0 ± 3.21) and Group III (126 ± 21), were higher, respectively, compared to the other groups. Moreover, the heavy metals, i.e., cadmium, lead, and copper, showed significant variation among the communities (*p*< 0.05), revealing their prominent role in maintaining communities’ structure and composition.

**Table 2:** Soil characteristics of different *N*. *officinale* dominated vegetation in the Swat River tributaries.

Factors	Groups					ANOVA	
	I		II	III	IV		
	M ± SE		M ± SE	M ± SE	M ± SE	*F*-value	*p*-value
Alt	915.28 ± 139		763.5 ± 50	629 ± 19.7	919 ± 83.4	2.178	0.11
Lat.	34.82 ± 0.099		34.7 ± 0.06	34.5 ± 0.1	34.8 ± 0.05	1.749	0.17
Long	72.15 ± 0.12		72.1 ± 0.04	71.9 ± 0.1	72.2 ± 0.07	1.589	0.21
AD	111.2 ± 32.1 ^d^		239.6 ± 10 ^a^	167.4 ± 41 ^bc^	192.6 ± 27 ^ab^	5.279	0.004
Clay	7.14 ± 0.5		14.6 ± 0.8	10 ± 2.28	8.22 ± 0.5	11.66	3.1×10^-5^
Silt	39.14 ± 1.8		50.07 ± 1.6	42 ± 3.94	51.5 ± 4.0	4.229	0.013
Sand	39.14 ± 3.6		43.2 ± 2.5	30.4 ± 2.7	50.6 ± 2.8	6.122	0.002
pH	7.9 ± 0.037		7.9 ± 0.04	7.72 ± 0.09	7.9 ± 0.04	3.919	0.017
EC	0.26 ± 0.02		0.25 ± 0.02	0.3 ± 0.05	0.26 ± 0.02	0.472	0.703
TDS	0.06 ± 0.003		0.09 ± 0.005	0.09 ± 0.01	0.08 ± 0.007	3.008	0.045
CC	6.71 ± 0.03		6.75 ± 0.0	6.75 ± 0.0	6.75 ± 0.00	1.323	0.285
OM	0.92 ± 0.02		0.93 ± 0.02	0.926 ± 0.07	0.97 ± 0.02	7.747	0.0005
N	0.046 ± 0.001		0.04 ± 0.002	0.04 ± 0.004	0.04 ± 0.003	0.252	0.858
P	22.941 ± 2		35.0 ± 3.21	28.03 ± 4.0	34.9 ± 3.15	4.440	0.010
K	74 ± 4.6		118.3 ± 17	126.8 ± 21	124.1 ± 16	2.945	0.048
WP	0.096 ± 0.005		0.09 ± 0.003	0.09 ± 0.008	0.08 ± 0.001	1.487	0.237
FC	0.25 ± 0.008		0.24 ± 0.005	0.25 ± 0.01	0.23 ± 0.006	0.951	0.428
BD	1.50 ± 0.03		1.49 ± 0.01	1.53 ± 0.06	1.55 ± 0.01	0.978	0.415
SP	0.43 ± 0.01		0.43 ± 0.006	0.42 ± 0.02	0.41 ± 0.004	0.988	0.411
AW	0.15 ± 0.005		0.14 ± 0.004	0.15 ± 0.01	0.14 ± 0.005	1.202	0.325
Cd	1.71 ± 0.32		3.22 ± 0.52	1.88 ± 0.5	3.7 ± 0.5	3.10	0.04
Pb	36.92 ± 6.3		70.88 ± 6.7	39.18 ± 7.5	68.35 ± 11	4.15	0.01
Cu	1.52 ± 0.17		5.04 ± 1.14	2.16 ± 0.12	3.16 ± 0.2	3.19	0.04
Zn	8.02 ± 0.86		6.59 ± 1.07	6.94 ± 0.89	6.56 ± 0.6	0.43	0.74

Alt, altitude; Lat, latitude; AD, aspect degree; pH, power of hydrogen; EC, electrical conductivity; M, Mean; SE, Standard error; ANOVA, Analysis of variance. Note: Different superscript indicate significant difference at P < 0.05. TDS, total dissolved salts; CC, calcium carbonate; OM, organic matter; N, nitrogen; P, phosphorus; K, potassium; WP, wilting point; FC, field capacity; SP, saturation point; BD, bulk density; AW, available water; Cd, cadmium; Pb, lead; Cu, copper; Zn, zinc.

The anthropogenic factors identified by respondents during the semi-structure interview showed non-significant *χ* ^2^ results between the Category “A” and Category “B” population, revealing that both the categories of factors affect the vegetation of *N. officinale*-dominated communities (**Table 3**). In addition, the lower cumulative variance percentage indicated that these anthropogenic factors equally contributed in disturbing vegetation groups. The quantitative anthropogenic factors (**Table 4**) vary across the communities coupled with IVI gradients; i.e., mining factor, cultivated fields percentage, and grazing intensity were higher in Group II and progressively decrease towards Group I via Groups III and IV (*F* = 3.56 and 7.61, respectively at *p*< 0.05). However, the reverse was true for over-harvesting and varied significantly, having *F* = 9.21 (*p*< 0.001). The diversity indices show increasing trends with increase in elevation. Similarly, the diversity indices were in reverse trends with *N. officinale* IVI; i.e., Group II has low species richness (3.33 ± 0.49) and higher IVI, while Group III with high species richness has low IVI. The Shannon–Wiener diversity index ranges from 0.97 ± 0.20 to 1.16 ± 0.24 and varies significantly (*p*< 0.05). Similarly, the species evenness index ranges from 0.62 ± 0.07 to 0.69 ± 0.05, showing significant variation (*p* > 0.05), indicating that the anthropogenic factors disturbed the group’s diversity.

**Table 3:** Anthropogenic disturbance semi-structure interview qualitative factor citation identified by respondents.

Factor	MF	CF	GI	OH	CV %
Category A	47 (49.4%)	33 (34.7%)	45 (47.3%)	70 (73.6%)	21.14
Category B	75 (81.5%)	30 (31.5%)	72 (78.25)	85 (92.35%)	10.65
*χ* ^2^-value	3.35	P-value	0.33	Non-significant	

Category A, random selected respondents; Category B, non-random selected respondents; MF, mining factor; CF, cultivation factor; GI, grazing intensity; OH, over-harvesting; CV, cumulative variance percentage.

**Table 4:** Anthropogenic factors affecting *Nasturtium officinale* communities.

Group	I	II	III	IV	*F*-value	*p*-value
Factor	M ± SE	M ± SE	M ± SE	M ± SE		
MF	15.72 ± 5.3 ^d^	42.08 ± 15.73 ^ab^	34 ± 24 ^bc^	40 ± 22 ^a^	3.56	0.02
CF	12.71 ± 20.70 ^b^	43.33 ± 17.62 ^a^	17 ± 8.36 ^c^	16 ± 3.94 ^c^	7.61	0.0006
GI	21.43 ± 6.26 ^d^	68.75 ± 12.63 ^a^	42 ± 25.8 ^c^	54.5 ± 19.5 ^b^	12.88	0.000014
OH	59.29 ± 14.26 ^a^	25.42 ± 9.15 ^d^	44 ± 21.9 ^b^	31.5 ± 15 ^c^	9.21	0.00019
S	6.71 ± 0.95 ^ab^	3.33 ± 0.49 ^d^	7 ± 2 ^a^	5.9 ± 0.87 ^bc^	25.3	0.0000021
H	1.04 ± 0.35 ^a^	1.16 ± 0.24 ^a^	1 ± 0.40 ^a^	0.97 ± 0.20 ^ab^	0.95	0.03
J	0.69 ± 0.05 ^a^	0.65 ± 0.13 ^bc^	0.67 ± 0.08 ^ab^	0.62 ± 0.07 ^cd^	3.89	0.03

MF, mining factor; CF, cultivation factor; GI, grazing intensity; OH, over-harvesting; S, species richness; H’, Shannon–Wiener diversity index; J, evenness index; different superscripts represent significant differences of the mean values.

### 3.4 Response of communities’ composition to environmental and anthropogenic factors

**Figure 5:**
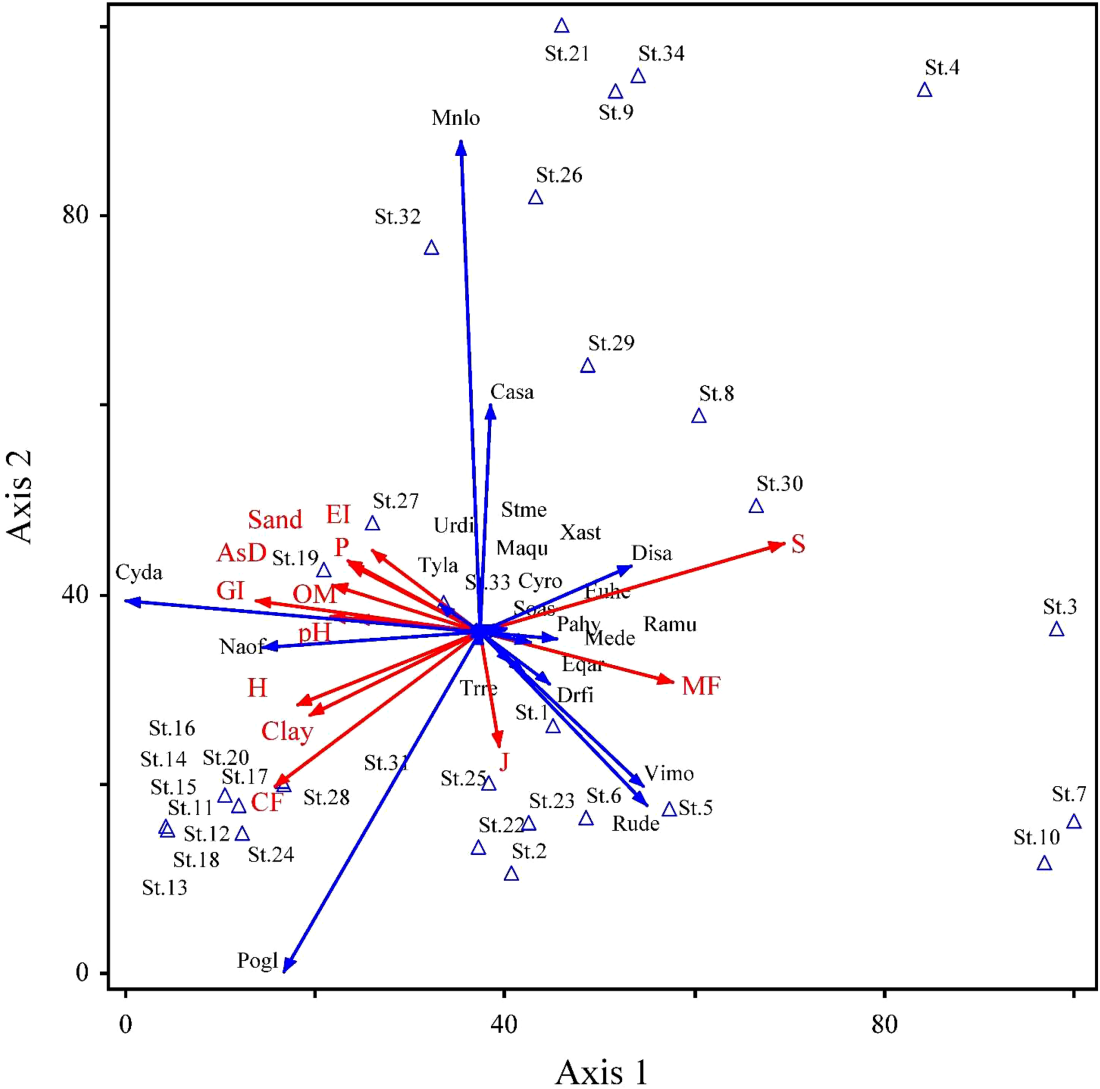
Projection of 22 species distributed in 34 stands with corresponding environmental variables on the 2D-RDA axis along the Swat River catchments and small tributaries. Importance values of species and 24 environmental variables were used in the analysis (for details, see **Tables 2**, **5**, **Table A1**). Plant species, and environmental and anthropogenic factors’ acronyms are the same as that in **Tables 1**–**4**.

In the RDA ordination (**Table 5**), the species–environment association was higher on the first two axes, contributing 44.8% of the overall variation (50.6%). Each of the 24 soil variables separately contributed to the overall ordination since none of the inflation factors had a score higher than 20.0. The Inter-set correlations, soil variables, like organic matter, clay, and pH, showed a significant positive correlation with the first axis (*r* = 0.51, 0.56, and 0.49, respectively). Similarly, silt, sand, and organic matter were significantly positively correlated on axis 2 (*r* = 0.32, 0.31, and 0.31 respectively). In contrast, sand percentage had a negative correlation (*r* = −0.25) on axis 3. Similarly, in anthropogenic variables, mining factor, cultivation factor, and grazing intensity showed positive correlation on axis 1 (*r* = 0.46, 0.57, and 0.67 respectively), while overexploitation showed negative correlation on axis 1 (*r* = −0.61). An unconstrained Monte Carlo permutation test (499 permutations) revealed that the *F*-ratio for the eigenvalue of axis 1 was significant (*p* = 0.05). The RDA ordination bi-plot demonstrated the correlation between the stands occupied on the right side and several soil characteristics, including organic matter, clay, sand, clay, phosphorus, pH, and aspect degree (**Figure 5**). On the other hand, electrical conductivity and calcium carbonate were associated with the remaining plant groups that filled the bi-plot’s left side. These factors also show higher biplot scores (**Table A3**).

**Table 5:** Axis summary statistics (number of canonical axes: 3 of a possible 22; total variance in the species data: 43.8).

Axis	Axis 1	Axis 2	Axis 3
Eigenvalue	4.892	2.395	2.358
Variance in species data			
% of variance explained	22.2	10.9	10.7
Cumulative % explained	22.2	33.1	43.8
Pearson Corr., Response-Pred.*	0.999	0.987	0.992
Kendall Corr., Response-Pred.	0.936	0.817	0.799

## 4 Discussion

The floristic composition shows a narrow spectrum of vegetation in the aquatic plant communities of *N. officinale* across the River Swat having 22 species, which are dominated by native species (77%) and non-native species (invasive, naturalized, and cultivated). Similarly, the range of life forms comprises annual herbaceous plants representative of 16 plant families. These spectrums show the physiognomy of the flora and vegetation, which is the result of all living activities combined with the environment, making it vital to comprehend the ecological foundation of vegetation of *N. officinale* (Amjad et al., 2016). The dominance of annual plant species in communities can bring community homogeneity that favors the invasion of foreign species (Milchunas et al., 1988; Tappeiner et al., 1991; Qian and Guo, 2010). The vegetative reproductive potential of *N. officinale* may also contribute to community homogenization, resulting in the formation of less diverse vegetation. Similarly, smaller herb species like *Xanthium strumarium, Mentha longifolia, Cynodon dactylon*, and *Cannabis sativa* may be advantageous over native species since they can more easily spread across the communities (Timsina et al., 2011). The future vegetation diversity will be at risk, due to this relocation, which ultimately brings communities’ disturbance. These results were contrary to what has been observed in other aquatic ecosystems; non-native species comprise a significant and substantially greater component of submerged aquatic plant communities (Santos et al., 2011; Du Toit et al., 2021), while in this case, *N. officinale*, a native invasive species, dominated the communities.

The cluster analysis segregated four unique vegetation groups, each dominated by *N. officinale* and have different co-dominant species. *Polygonum glabrum* was the co-dominant species in Group I, *Cynodon dactylon* in Group II, *Digitaria sanguinalis* in Group III, and *Mentha longfolia* in Group IV. Similarly, a study was conducted on the phytosociology of *Sedum brevifolium* and found six plant groups (Nabi et al., 2012), while Ali et al. (2022) reported two plant communities across the wetlands of Panjkora River in district Dir Lower. At the sample sites, *N. officinale* was often found with several associated species, including *Polygonum glabrum*, *Cynodon dactylon*, and *Digitaria sanguinalis*. The same environmental factors and nutritional requirements might cause their communities’ association, as reported by Khan (2012); Khan et al. (2014); Muhammad et al. (2016); Nasrullah et al. (2015), and Shah and Hussain (2009).

The soil characteristics revealed that the calcareous nature accounted for roughly 99% of the growth of *N. officinale*. The calcareous soils with high potassium, nitrogen, and low phosphorus concentrations range from sandy loam to loamy silty texture, resulting in gregarious communities, as that reported in the region by Ullah et al. (2021) and Ullah et al. (2022). The smaller communities having high density and cover in terms of species composition may be attributed to two factors, i.e., the communities are new to the region and the potential of *N. officinale* to regenerate (Ali et al., 2022). Our results were contrasted to that reported by Khan et al. (2020), who reported that seedlings and juvenile plants grow quicker than mature plants, resulting in a decreased density. However, these results complied with Hitimana et al. (2004), where the high density is linked to the plant species’ capacity for regeneration. The community structure and composition may change due to man-made activities, intra- and inter-specific competition, and the pattern of regeneration (Faridah-Hanum et al., 2012). These soils also allow water and air to move through them, letting roots penetrate more readily, providing nutrients and clay aggregation stability (Zimmermann, 1991). Certain invasive generalist species, such as *C. sativa*, *X. strumarium*, *and C. dactylon*, pioneer species of disturbed vegetation, were dominant in the communities (Iqbal et al., 2021). *N. officinale* probably establishes itself as the dominant species in wetlands in Khyber Pakhtunkhwa due to these environmental factors that favor their propagation and growth. In addition, the vegetation decomposition and microbial activities in the root zone may further add soil nutrients that add to the fertility of soil and resulting in the establishment and propagation of a particular community in an area (Bargali et al., 2018).

As expected, the anthropogenic variables substantially disrupted the communities’ structure, favoring non-native species invasion (Geiger and McPherson, 2005; Bhattarai et al., 2014). The study assessed mining factor, grazing pressure, cultivated fields, and over-harvesting to determine how these factors relate to the disturbance of *N. officinale* communities. However, these factors vary significantly along IVI gradients, showing that they are linked to invasive species and community disturbance. The sites of Group II had greater grazing intensity, agricultural activities or areas occupied by cultivated fields, and mining factors, making the populations more susceptible to invasion, as reported by Chhogyel et al. (2021) and Pretto et al. (2010). This could be due to the easy transportation of the alien species propagules in such areas. According to McDougall et al. (2005) and Sjödin et al. (2008), grazing pressure and mining factor disturbed communities by generating unoccupied niches for alien propagules, enabling the plant to invade the vacant habitats. Similarly, sand extraction from aquatic environments, like rivers and coasts, provides an ecosystem service (Grizzetti et al., 2019). Sand is primarily used in construction because concrete contains 75% sand (Jnr et al., 2018). All this equates to approximately 200 tons of sand for a home, 30,000 tons for every kilometer of roadway, and an astounding 12 million tons of sand for a nuclear power station (Ludacer, 2018; Rentier, and Cammeraat, 2022). This tremendous sand demand has made mining a global environmental concern that disturbed community structure (Asabonga et al., 2017). This mining operation caused habitat fragmentation that continuously disturbed these communities in the river bed and char-land areas.

RDA of vegetation–soil interactions revealed that organic matter, silt, sand, clay, phosphorus, potassium, pH, latitude, longitude, aspect degree, wilting point, field capacity, and available water were the key factors controlling the distribution of vegetation in the region. The role of organic carbon in soil fertility is well known and reported by many authors like Kononova (2013) and Rao et al. (2017). Similarly, increase in soil organic matter content results from the breakdown of plant residues, lower salt toxicity from dissolved potassium and calcium brought by rainwater (Taiz and Zeiger, 2002), and increasing vegetation diversity (Lyons et al., 2005). Moreover, many studies like Ali et al. (2022); Ullah et al. (2022), and Ullah et al. (2021) in the same region reported the significance of soil organic matter, nitrogen, and soil texture in sustaining plant communities. Similar research on the influence of surface sediment percentages of various size classes on the geographical distribution of soil moisture was reported by Dasti and Agnew (1994). In addition, many other factors, such as precipitation, rain, and flood, increase the percentages of silt and clay in the soil texture that play an important role in shaping plant communities (Duniway et al., 2010). The most significant factors closely related to the dispersion of vegetation communities are nutritional status, electrical conductivity, soil texture, slope, and aspect (Tavili and Jafari, 2009). Our results were also in agreement with those of Zare et al. (2011), who reported that the distribution of plants is influenced by slope, aspect elevation, soil texture, lime content, soil moisture, available nitrogen, potassium, and organic matter, and Bargali et al. (2018), revealing the importance of soil organic matter and nitrogen in maintaining plant communities.

## 5 Conclusions

The study concluded that topography and spatial variability influenced the *N. officinale* vegetation’s distribution in the area. In addition, the anthropogenic factors qualitative and quantitative intensity have the most prominent role in disturbing the communities. In addition, it has been shown that the spread of *N. officinale* vegetation is more strongly correlated with physical environmental factors and substrate characteristics that affect water availability. The disturbance in communities can be minimized by land use to change the water conditions in wetlands directly or indirectly by draining them to make the surface drier and by introducing plant species like *N*. *officinale* that are tolerant to flooding and produce more crops and feed. In addition, non-wetland species swiftly builds up peaty soils in the wetlands, elevating the surface above the water building diverse communities. However, the presence of non-native species may subject the communities to unexpected changes in vegetation; therefore, proper management of non-native invasive species should be considered for future perspective.

## Data availability statement

The original contributions presented in the study are included in the article/**Supplementary Material**. Further inquiries can be directed to the corresponding author.

## Author contributions

NK and RU conceptualized this research, conducted the field and laboratory experiments, and compiled and analyzed the data; NK and RU wrote the original draft of the manuscript; MO and MA-M supervised the experiments; IS, H-AH, and TA contributed to data analysis; and HA helped in the review and edited the initial draft of the manuscript; all authors read and approved the manuscript.
