# Supplementary material for: Environmental and anthropogenic drivers of watercress (Nasturtium officinale) communities in char-lands and water channels across the Swat River Basin: implication for conservation planning
Source: Front Plant Sci. 2023 Sep 27;14:1225030. doi: 10.3389/fpls.2023.1225030 (PMC10569500; doi:10.3389/fpls.2023.1225030)
Supplement: Supplementary file 1 [file Table_1.docx]

**Table A 1:** Taxonomic characteristics of different plant species associated with *Nasturtium officinale*

| Species Binomials | BC | Family | HB | LC | SP | AH |
| --- | --- | --- | --- | --- | --- | --- |
| *Nasturtium officinale* W.T. Aiton | Naof | Brassicaceae | H | P | Nt | E/F |
| *Cannabis sativa* L*.* | Casa | Cannabaceae | H | A | Nt | E |
| *Cynodon dactylon* (L.) Pers | Cyda | Poaceae | H | P | Nt | E/F |
| *Cyperus rotundus* L. | Cyro | Cyperaceae | H | P | Nt | E |
| *Digitaria sanguinalis* (L.) scop. | Disa | Poaceae | H | A | Nt | E/F |
| *Dryopteris filix-mas* (L.) Schott | Drfi | Dryopteridaceae | H | A | Nt | E |
| *Equisetum arvense* L. | Eqar | [Equisetaceae](https://plants.usda.gov/java/ClassificationServlet?source=display&classid=Equisetaceae) | H | P | Nt | E |
| *Euphorbia helioscopia* L. | Euhe | Euphorbiaceae | H | A | Nt | E |
| *Marsilea quadrifolia*L. | Maqu | Marsileaceae | H | A | Nt | E/F |
| *Medicago denticulata* Willd. | Mede | Fabaceae | H | A | I | E |
| *Mentha longifolia* (L.) Huds. | Melo | Lamiaceae | H | P | Nt | E |
| *Parthenium hysterophorus* L. | Pahy | Asteraceae | H | A | I | E |
| *Polygonum glabrum* Willd. | Pogl | Polygonaceae | H | A | Nt | E |
| *Ranunculus muricatus* L., | Ramu | Ranunculaceae | H | A | Nt | E |
| *Rumex dentatus* L. | Rude | Polygonaceae | H | A | Nt | E |
| *Sonchus asper* (L.) Hill | Soas | Asteraceae | H | A | Na | E |
| *Stellaria media* (L.) Vill. | Stme | Caryophyllaceae | H | A/P | Cm | E |
| *Trifolium repens* L. | Trre | Poaceae | H | P | Nt | E |
| *Typha latifolia* L., | Tyla | Typhaceae | H | P | Nt | E |
| *Urtica dioica* L. | Urdi | Urticaceae | h | P | Nt | E |
| *Vicia monantha* Retz., | Vimo | Fabaceae | h | A | Nt | E |
| *Xanthium strumarium* L. | Xast | Asteraceae | h | A | I | E |

**Note:**BC (binomial code); HB (habit); H (herb); LC (life cycle); A (annual); P (perennial); A/P (annual/perennial); SP (status in Pakistan); Cm (cosmopolitan); Nt (native); I (invasive); Na (naturalize); AH (aquatic habitat); E (emergent); E/F (emerged/floating).

**Table 2:** Mean values of Density (individuals /hectare) and Cover/hectare (m^2^) of *Nasturtium officinale* and different associated plant species.

| Species Binomial |  | Density | | | | Cover | | | | |
| --- | --- | --- | --- | --- | --- | --- | --- | --- | --- | --- |
|  |  | Group-I | Group-II | Group-III | Group-IV | Group-I | Group-II | Group-III | | Group-IV |
|  | Acronyms | M±SE | M±SE | M±SE | M±SE | M±SE | M±SE | | M±SE | M±SE |
| *Nasturtium officinale* W.T. Aiton | Naof | 46243±4119 | 57094±2469 | 47555±7702 | 62139±3140 | 4994±359 | 7079±211 | | 5980±255 | 6625±115 |
| *Cannabis sativa* L*.* | Casa | *- | *- | 74±74 | 370±213 | *- | *- | | 10±10 | 33.3±22.2 |
| *Cynodon dactylon* (L.) Pers | Cyda | 18730±2316 | 13162±1939 | 1629±1629 | 11851±2051 | 1993±256 | 1586±234 | | 128±128 | 1258±211 |
| *Cyperus rotundus* L. | Cyro | *- | *- | 4222±4222 | *- | *- | *- | | 331±331 | *- |
| *Digitaria sanguinalis* (L.) scop. | Disa | 5343±2719 | 3219±2182 | 10222±3723 | 9300±2009 | 282±188 | 296±200 | | 1584±371 | 842±204 |
| *Dryopteris filix-mas* (L.) Schott | Drfi | *- | *- | 222±222 | *- | *- | *- | | 46±46 | *- |
| *Equisetum arvense* L. | Eqar | 740±740 | *- | 1555±996 | *- | 59±59 | *- | | 291±182 | *- |
| *Euphorbia helioscopia* L. | Euhe | 52±52 | *- | 74±74 | 41±41 | 12±12 | *- | | 10±10 | 3.7±3.7 |
| *Marsilea quadrifolia*L. | Maqu | *- | *- | *- | 1316±1316 | *- | *- | | *- | 189±189 |
| *Medicago denticulata* Willd. | Mede | 370±242 | *- | 222±222 | *- | 82±53 | *- | | 31±31 | *- |
| *Mentha longifolia* (L.) Huds. | Melo | 264±209 | *- | 1259±934 | 6213±2681 | 44±36 | *- | | 162±133 | 345±120 |
| *Parthenium hysterophorus* L. | Pahy | *- | *- | 2518±1357 | *- | *- | *- | | 334±170 | *- |
| *Polygonum glabrum* Willd. | Pogl | 8201±1377 | 7635±1843 | 2666±1362 | 5349±1498 | 965±171 | 986±219 | | 365±130 | 545±140 |
| *Ranunculus muricatus* L., | Ramu | 1481±685 | *- | 296±215 | 452±211 | 177±79 | *- | | 37±24 | 52±25 |
| *Rumex dentatus* L. | Rude | 3809±834 | *- | 444±272 | 205±139 | 459±114 | *- | | 65±42 | 23±15 |
| *Sonchus asper* (L.) Hill | Soas | 264±264 | *- | *- | *- | 57±57 | *- | | *- | *- |
| *Stellaria media* (L.) Vill. | Stme | 211±211 | *- | 2666±1777 | *- | 301±263 | *- | | 126±126 | *- |
| *Trifolium repens* L. | Trre | *- | 341±341 | *- | *- | *- | 48±48 | | *- | *- |
| *Typha latifolia* L., | Tyla | *- | 626±626 | *- | *- | *- | 74±74 | | *- | *- |
| *Urtica dioica* L. | Urdi | 370±370 | *- | *- | *- | 54±54 | *- | | *- | *- |
| *Vicia monantha* Retz., | Vimo | 1058±701 | *- | 2962±1245 | *- | 97±63 | *- | | 480±215 | *- |
| *Xanthium strumarium* L. | Xast | *- | 227±98 | *- | *- | *- | 6.26±6.26 | | *- | *- |
| *Total* |  | 14 | 7 | 16 | 10 | 14 | 7 | | 16 | 10 |

Note: M (mean value); SE (standard error); *- (species absence in group)

**Table A 3:** Specie relation with Environmental, soil and Anthropogenic factors assessed via RDA analysis in different *Nasturtium officinale* dominated stands

|  | Correlations | | | Biplot scores | | |
| --- | --- | --- | --- | --- | --- | --- |
| Variable | Axis 1 | Axis 2 | Axis 3 | Axis 1 | Axis 2 | Axis 3 |
| Alt. | 0.202 | 0.207 | 0.121 | 0.594 | 0.427 | 0.247 |
| Lat. | 0.364 | 0.251 | 0.118 | 1.07 | 0.516 | 0.241 |
| Long | 0.331 | 0.301 | 0.173 | 0.975 | 0.621 | 0.353 |
| As.D. | 0.307 | 0.032 | -0.082 | 0.902 | 0.066 | -0.167 |
| Clay | 0.569 | -0.05 | 0.102 | 1.676 | -0.103 | 0.209 |
| Slit | 0.34 | 0.328 | 0.023 | 1.001 | 0.676 | 0.047 |
| Sand | 0.276 | 0.311 | -0.256 | 0.812 | 0.641 | -0.523 |
| pH | 0.49 | 0.143 | -0.005 | 1.442 | 0.295 | -0.01 |
| EC | -0.331 | 0.012 | 0.056 | -0.973 | 0.024 | 0.115 |
| TDS | -0.201 | 0.104 | 0.016 | -0.592 | 0.214 | 0.032 |
| CC | 0.133 | 0.228 | 0.858 | 0.391 | 0.47 | 1.754 |
| OM | 0.516 | 0.308 | -0.036 | 1.519 | 0.633 | -0.073 |
| N | -0.077 | 0.206 | -0.091 | -0.226 | 0.423 | -0.187 |
| P | 0.397 | 0.208 | -0.081 | 1.167 | 0.429 | -0.166 |
| K | 0.287 | 0.204 | -0.001 | 0.844 | 0.42 | -0.003 |
| WP | 0.367 | -0.091 | 0.195 | 1.079 | -0.186 | 0.398 |
| FC | 0.091 | -0.179 | 0.308 | 0.267 | -0.368 | 0.63 |
| BD | -0.352 | 0.026 | -0.201 | -1.035 | 0.054 | -0.41 |
| SP | 0.353 | -0.026 | 0.203 | 1.038 | -0.053 | 0.414 |
| AW | -0.131 | -0.184 | 0.294 | -0.386 | -0.379 | 0.6 |
| Cd | 0.017 | -0.137 | -0.233 | 0.05 | -0.283 | -0.476 |
| Pb | -0.222 | -0.08 | 0.129 | -0.654 | -0.164 | 0.264 |
| Cu | 0.221 | -0.063 | 0.004 | 0.65 | -0.129 | 0.009 |
| Zn | -0.022 | 0.045 | 0.159 | -0.065 | 0.092 | 0.326 |
| S | -0.899 | -0.033 | -0.08 | -2.646 | -0.068 | -0.163 |
| H | 0.58 | -0.046 | -0.03 | 1.706 | -0.094 | -0.061 |
| J | -0.032 | -0.248 | 0.037 | -0.095 | -0.51 | 0.076 |
| MF | 0.468 | 0.335 | 0.128 | 1.376 | 0.69 | 0.261 |
| CF | 0.571 | -0.312 | 0.099 | 1.679 | -0.642 | 0.203 |
| GI | 0.678 | 0.252 | 0.098 | 1.995 | 0.518 | 0.2 |
| OE | -0.618 | -0.324 | -0.073 | -1.819 | -0.667 | -0.148 |

Note: The acronyms are same as that in Table 2, 3 and 4
